# Supplementary material for: Humoral Immune Response in IBD Patients Three and Six Months after Vaccination with the SARS-CoV-2 mRNA Vaccines mRNA-1273 and BNT162b2
Source: Biomedicines. 2022 Jan 13;10(1):171. doi: 10.3390/biomedicines10010171 (PMC8774019; doi:10.3390/biomedicines10010171)
Supplement: Supplementary file 1 [file biomedicines-10-00171-s001.zip › biomedicines-1542614-supplementary.pdf]

Supplementary Table S1: Laboratory values of IBD patients before the first vaccination, before the second vaccination and three months after the second vaccination. Division of patients according to applied immunosuppressive therapy. Patients on therapy with cortisone, tofacitinib, certolizumab, golimumab, risankizumab, etrolizumab, cellcept were grouped under "others". IBD, inflammatory bowel disease; IQR, interquartile range; AST, aspartate aminotransferase; CRP, C-reactive protein.

**IBD Patients**	**Before 1st Vacci.**	**Before 2nd Vacci.**	**3 Months after 2nd Vacci.**
Leukocytes (x10^9/L), median (IQR)	7.2 (6.1-8.7)	7.3 (6.1-8.6)	7.5 (6-9)
Creatinine (mg/dL), median (IQR)	0.8 (0.7-0.9)	0.8 (0.7-0.9)	0.9 (0.8-1.1)
Bilirubin (mg/dL), median (IQR)	0.4 (0.3-0.7)	0.3 (0.2-0.6)	0.4 (0.3-0.5)
AST (U/L), median (IQR)	17 (13-23)	20 (15-25)	20 (13-31)
CRP (mg/dL), median (IQR)	0.5 (0.5-0.8)	0.5 (0.5-0.8)	0.5 (0.5-0.5)
Ferritin (µg/L), median (IQR)	27 (12-66)	35 (16-76)	51 (16-88)

Supplementary Table S2: Patient characteristics of IBD patients and healthy controls. IBD, inflammatory bowel disease; IQR, interquartile range; BMI, body mass index; SARS-CoV-2, severe acute respiratory syndrome coronavirus 2.

		**IBD-Patients (n = 95)**	**Controls (n = 38)**	***p*-Value**
Patient characteristics	Age, years median (IQR)	46 (33-55)	33 (28-48)	0.09
	Sex, male (%)	50 (53)	11 (29)	<0.001
	Death (abs.)	0	0	1
	BMI (kg/m^2), median (IQR)	24.8 (21.6-28.4)	23.7 (21.7-27.2)	0.171
SARS-CoV-2 vaccine	mRNA-1273 (%)	6 (6)	31 (82)	<0.001
	BNT162b2 (%)	89 (94)	7 (18)	<0.001
